# Short Communication: An evaluation of M 1001 Compur for the enzymic determination of HDL Cholesterol

**DOI:** 10.1155/S1463924680000636

**Published:** 1980

**Authors:** Claus C. Heuck, Günter Schlierf

**Affiliations:** Klinisches Institut für Herzinfarktforschung 69 Heidelberg Bergheimer Str. 58 Germany


### Short Communication

### An evaluation of M 1001 Compur for the enzymic determination of HDL - Cholesterol

**Claus C. Heuck and Günter Schlierf**

*Klinisches Institut für Herzinfarktforschung, 69 Heidelberg, Bergheimer Str. 58, West Germany.*

### Introduction

A quick and simple method for the enzymic determination of serum cholesterol has recently become available using a portable minicentrifuge and mini-photometer M 1001 (Compur, München, West Germany) [1]. The mini-photometer is battery operated and measures at a fixed wave length of 510 nm. Cholesterol is determined with the cholesterol esterase/cholesterol oxidase/phenylaminophenazone method. The reagent is supplied by the manufacturer in pre-prepared cuvettes [1]. Since this system requires only a very small amount of sample and reagent, it is valuable for the direct determination of cholesterol in high density lipoprotein (HDL) in human and animal serum samples, when only small volume samples can be obtained and major laboratory facilities are not available.

### Materials and methods

Serum samples from normolipemic volunteers and subjects with hyperlipoproteinemia were obtained 14 hours after the last food intake. The samples were routinely analysed for total cholesterol and triglyceride concentrations in serum [2], and HDL cholesterol isolated by ultracentrifugation and heparin/manganese chloride precipitation [3]. Serum total cholesterol was also measured enzymatically with the mini-photometer according to the recommendations of the manufacturer.

For the enzymic determination of cholesterol in HDL using the mini-photometer 100 $\mu$l of fresh or frozen serum was mixed with 20 $\mu$l of a solution containing 12.8 mmol/1 phosphotungstic acid (PTA) and 0.5 mol/1 of magnesium chloride ($MgCl_2$) adjusted with 1 m NaOH to pH 7.5. After 10 minutes the mixture was centrifuged at 6000 rpm in a laboratory centrifuge in Eppendorf polyethlene-tubes to remove lipoprotein-polyanion precipitates. Ten microlitres of the supernatant solution were added to the photometer cuvettes (Compur, München, West Germany) containing 600 $\mu$l of the appropriate enzyme solution. Photometric measurements were made with the mini-photometer. The absorbance was read prior to the addition of the probe to the reagent and after 15 minutes of reaction time. As the scale of the photometer is calibrated in mmol/1 the obtained value for $\Delta E$ was multiplied by a factor 0.6 to calculate the concentration in mmol/1 and by 23.22 for calculations in mg/100 ml of HDL-cholesterol.

The factors in the calculation take into account the dilution of the sample volume by the polyanion reagent and the different volume (10 $\mu$l) added to the enzyme solution for the determination of HDL-cholesterol compared to that for the determination of serum cholesterol from 5 $\mu$l of serum sample, for which the scale of the photometer is calibrated by the manufacturer.

The selectivity of precipitating very low density lipoprotein (VLDL) and low density lipoprotein (LDL with PTA/$MgCl_2$ was studied by immuno electrophoresis [5, 6]. The agarose plates contained either anti-apolipoprotein B or anti-apolipoprotein $A_1$. The precision of the determination of the HDL-cholesterol was estimated with unfrozen

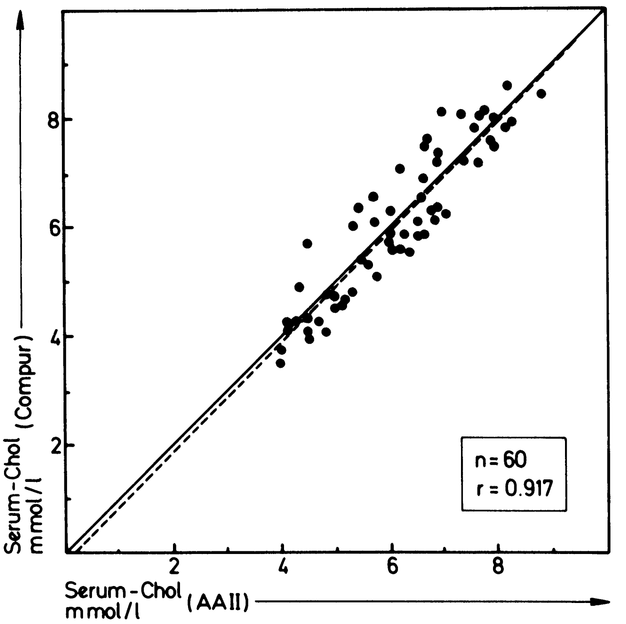

*Figure 1a. Comparison of the determination of total cholesterol in serum using an AutoAnalyzer (AAII) and the enzymic technique with the Compur mini-photometer (n:60). The serum samples were obtained from normolipemic and hyperlipoproteinemic subjects (type IIa IIb, and III and IV). The linear regression line is shown dotted (slope: 1.01; y-intercept = –0.16 mmol/1).*

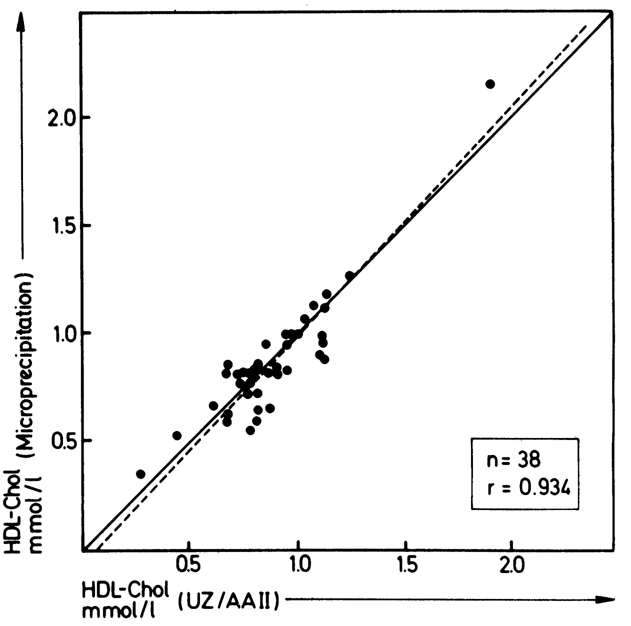

*Figure 1b. Comparison of the determination of HDL-cholesterol in fresh human serum samples by the routine procedure [3] and by the microprecipitation procedure (n:38). The linear regression line is shown dotted (slope: 1.03; y-intercept = –0.037 mmol/1).*

human serum samples and with a frozen rat serum sample, sixteen determinations being made on each.

During an experimental study, 0.4 ml of blood was obtained from male Sprague-Dawley rats (180–200 gm weight) by puncture of the ophtalmic plexus. Each rat was punctured four times at intervals of 24 hours. The serum samples were frozen for one week prior to the determination of HDL-cholesterol concentration according to the method described above.

### Results and discussion

The enzymic determination of serum cholesterol using the mini-photometer system correlates well (r: 0.917) with the semi-automated AutoAnalyzer method within the range of 4 mmol/l and 9 mmol/l of cholesterol (Figure 1a). The estimations were made with three different batches of cuvettes. Using only one batch the correlation was even higher (r: 0.982; n = 20 serum samples). The correlation is of similar degree (r: 0.934) for HDL-cholesterol determined by the routine procedure after fractionation with the ultracentrifuge and by measurement of supernatant after polyanion precipitation with the mini photometer (Figure 1b).

The precipitation of VLDL and LDL by $PTA/MgCl_2$ under the established conditions was complete, as indicated by rocket immuno electrophoresis. No apolipoprotein B was detected in the supernatant solution (Figure 2). In contrast, for apolipoprotein $A_1$ which is transported mainly in HDL, the recovery was 90%. This confirms the previous observations on the selectivity of polyanion interaction with apolipoprotein B transporting lipoproteins [5]. With other polyanions such as dextransulfate/$MgCl_2$ or heparin/$MnCl_2$ statistically similar results were obtained. However PTA/$MgCl_2$ was preferred since it is the most stable and cheapest reagent commercially available. The coefficient of variation ($c_V$) for 16 estimations of unfrozen human serum was 6.5 (mean: 1.22 mmol/1± 0.079 SD HDL-cholesterol), 8.6 for estimations made in a similar series over a 14 day period and 5.41 (mean: 1.03 mmol/1± 0.056 SD HDL-cholesterol) for 16 determinations on frozen rat serum. The better result for rat serum is probably due to the low concentration of VLDL and the virtual absence of LDL in serum from fasted animals. The data obtained in the longitudinal study on rats (Table 1) indicate that the procedure may be used for determination on frozen serum samples.

The microphotometric method has the advantage of being less laborious than methods involving ultracentrifugation [3] or electrophoresis/gaschromatography [6]. It may be used for field studies and ambulatory surveys, since the instrument is easily transported and small samples of blood may be obtained from the ear or finger tip. It is however limited to serum samples with triglyceride concentrations lower than 4.5 mmol/1. Above this level the precision of measurement decreases.

ACKNOWLEDGEMENT

The authors wish to thank Miss K. Frech and Mrs. I. Erbe for skilled technical assistance.

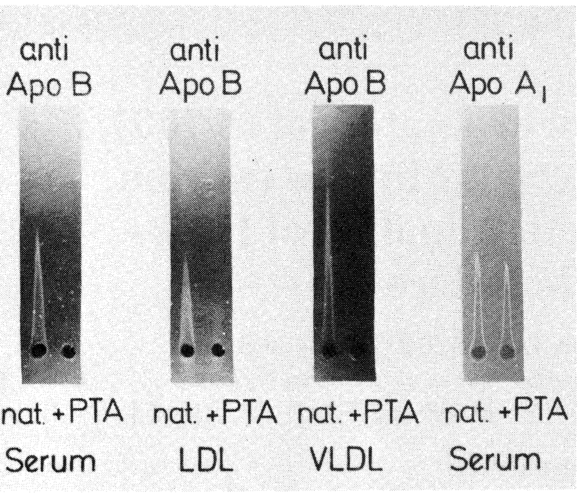

*Figure 2. Rocket immunoelectrophoresis of serum, isolated VLDL and isolated LDL-solutions with apolipoprotein B and apolipoprotein $A_1$ antiserum. Each illustration shows the results from the untreated sample and after treatment with phosphotungstic acid ($PTA/MgCl_2$).*

**Table 1. Determination of cholesterol and HDL in serum from 10 rats (180-200 gm weight). (From each animal 0.5 ml of blood was obtained on four occasions during a period of 72 hours.)**

| Time (hours) | 0 | 24 | 48 | 72 |
|---|---|---|---|---|
| serum cholesterol (mmol/1) | 1.37 ± 0.4 | 1.34 ± 0.14 | 1.30 ± 0.14 | 1.29 ± 0.12 |
| HDL–cholesterol (mmol/1) | 1.05 ± 0.14 | 1.03 ± 0.10 | 1.00 ± 0.14 | 1.03 ± 0.10 |

REFERENCES

[1] Frank, G. and Wehling, K., Abstracts of Papers and Posters 3rd European Congress on Clinical Chemistry, June 1979, Brighton, England.

[2] Technicon, Clinical Method, No 24, 1972 Technicon Instruments, Tarry Town, N.Y., USA.

[3] Manual of Laboratory Operations, Lipid Research, Clinics Program, 1974, DHEW Publication No. (NIH) 75–628 Bethesda, Md. 20014, USA.

[4] Lopes-Virella, M. F., Stone, P., Ellis, S. and Caldwell, J. A. *Clinical Chemistry,* 1977, **23**, 882.

[5] Kostner, G. M., in 'Handbuch der Inneren Medizin' Vol. 7 Fettstoffwechselstörungen, Ed. Schettler, G. Greten, H. Schlierf, G., and Seidel, D., 1976, Springer Verlag, Berlin, Heidelberg, New York, p 25.

[6] Heuck, C. C., Erbe, I., Wirth, A. and Schlierf, G., *Clinica Chimica Acta,* 1979, **98**, 195-199.

[7] Heuck, C. C., Nothhelfer, A., Raetzer, H. and Schlierf, G. *Journal of Lipid Research*, 1977, **18**, 259.

